# Environmental and Physiological Factors Affecting High-Throughput Measurements of Bacterial Growth

**DOI:** 10.1128/mBio.01378-20

**Published:** 2020-10-20

**Authors:** Esha Atolia, Spencer Cesar, Heidi A. Arjes, Manohary Rajendram, Handuo Shi, Benjamin D. Knapp, Somya Khare, Andrés Aranda-Díaz, Richard E. Lenski, Kerwyn Casey Huang

**Affiliations:** a Department of Chemical and Systems Biology, Stanford University School of Medicine, Stanford, California, USA; b Department of Microbiology and Immunology, Stanford University School of Medicine, Stanford, California, USA; c Department of Bioengineering, Stanford University, Stanford, California, USA; d Biophysics Program, Stanford University School of Medicine, Stanford, California, USA; e Department of Microbiology and Molecular Genetics, Michigan State University, East Lansing, Michigan, USA; f BEACON Center for the Study of Evolution in Action, Michigan State University, East Lansing, Michigan, USA; g Chan Zuckerberg Biohub, San Francisco, California, USA; University of Utah

**Keywords:** density-dependent growth, glycerol, lag phase, long-term evolution experiments, teichoic acids

## Abstract

Bacterial growth under nutrient-rich and starvation conditions is intrinsically tied to the environmental history and physiological state of the population. While high-throughput technologies have enabled rapid analyses of mutant libraries, technical and biological challenges complicate data collection and interpretation. Here, we present a framework for the execution and analysis of growth measurements with improved accuracy over that of standard approaches. Using this framework, we demonstrate key biological insights that emerge from consideration of culturing conditions and history. We determined that quantification of the background absorbance in each well of a multiwell plate is critical for accurate measurements of maximal growth rate. Using mathematical modeling, we demonstrated that maximal growth rate is dependent on initial cell density, which distorts comparisons across strains with variable lag properties. We established a multiple-passage protocol that alleviates the substantial effects of glycerol on growth in carbon-poor media, and we tracked growth rate-mediated fitness increases observed during a long-term evolution of Escherichia coli in low glucose concentrations. Finally, we showed that growth of Bacillus subtilis in the presence of glycerol induces a long lag in the next passage due to inhibition of a large fraction of the population. Transposon mutagenesis linked this phenotype to the incorporation of glycerol into lipoteichoic acids, revealing a new role for these envelope components in resuming growth after starvation. Together, our investigations underscore the complex physiology of bacteria during bulk passaging and the importance of robust strategies to understand and quantify growth.

## Abstract (precis)

How starved bacteria adapt and multiply under replete nutrient conditions is intimately linked to their history of previous growth, their physiological state, and the surrounding environment. While automated equipment has enabled high-throughput growth measurements, data interpretation and knowledge gaps regarding the determinants of growth kinetics complicate comparisons between strains. Here, we present a framework for growth measurements that improves accuracy and attenuates the effects of growth history. We determined that background absorbance quantification and multiple passaging cycles allow for accurate growth rate measurements even in carbon-poor media, which we used to reveal growth-rate increases during long-term laboratory evolution of Escherichia coli. Using mathematical modeling, we showed that maximum growth rate depends on initial cell density. Finally, we demonstrated that growth of Bacillus subtilis with glycerol inhibits the future growth of most of the population, due to lipoteichoic acid synthesis. These studies highlight the challenges of accurate quantification of bacterial growth behaviors.

## INTRODUCTION

Precise growth measurements are fundamental to our understanding of bacterial physiology and its regulation. While some bacterial species are among the fastest-growing organisms on the planet, others grow imperceptibly slowly, with doubling times ranging from ∼7 min (1) to thousands of years (2). Although the need for rapid growth may drive selection in some cases, many bacteria live in complex natural environments that are often stressful and nutrient limited (3). In many environments, such as the mammalian gut, leaf litter in soil, and whale falls in the ocean, food is provided only periodically; hence, bacteria experience cycles of feast and famine. Thus, the transition from starvation to rapid growth can also act as an important selective pressure in evolution (4–6).

**FIG 1:**
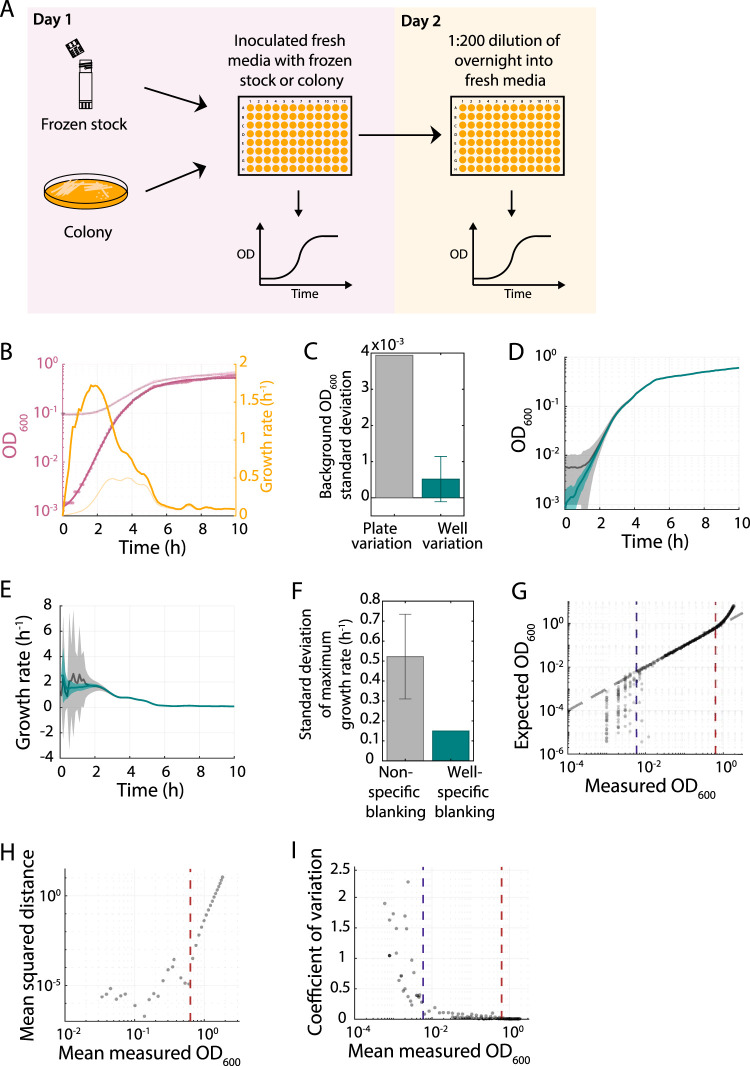
Well-specific blanking and establishment of the spectrophotometer limit of detection are critical for accurate measurements of population growth rate. (A) Schematic of a classical experimental setup to measure bacterial growth. (B) Raw absorbance values from a typical E. coli MG1655 growth curve (light purple curve) increased starting from just above the background absorbance of the well plus media (∼0.08). Subtracting the background OD resulted in absorbance values (dark purple circles) that indicate substantially different growth kinetics, which are well fit by a Gompertz function (dark purple curve). With background subtraction, the maximum growth rate was 3.7-fold higher and occured ∼60 min earlier (dark yellow curve) than without subtraction (light yellow curve). (C) The variation in the OD of a cell-free well containing only medium (gray) was much higher across a 96-well plate than the mean variation across multiple readings from a single well (teal). (D) The variation in E. coli growth curves across a 96-well plate when blanking with the cell-free absorbance of each well (teal) was much lower than when blanking with the background absorbance of a random well (black). Shaded regions represent ±1 standard deviation. (E) Instantaneous growth rates computed from growth curves in panel B were much less variable for well-specific blank subtraction (teal) than for blanking with a random well (black). (F) Well-specific blank subtraction (teal) dramatically decreased the standard deviation in the estimate of maximum growth rate compared to that with subtraction of the blank from a randomly selected well in a 96-well plate (gray). (G) A dilution series from a culture with a known OD can be used to calibrate OD readings in order to establish the range of linearity and the limit of detection of a plate reader. Blue and red dotted lines represent the linear range of detection; even above the linear range, the variability was low. Grey dashed line is *y* = *x*. (H) The mean squared distance from the line *x *=* y* in panel D increased sharply above the red dotted line, justifying its definition as the upper limit of the range of linearity. (I) The coefficient of variation (CV; mean/standard deviation) of OD values in panel D increased sharply below the blue dotted line, justifying its definition as the lower limit of detection. Given the low CV above the linear range (above the red dotted line), it is possible to accurately measure up to an actual OD of ∼3.5 through correction based on the calibration in panel G. The red dotted line represents the upper limit of the linear range.

A classical batch laboratory assay that encompasses all phases of growth involves the initial overnight growth of a liquid culture, from either a frozen stock or a colony, which is then used to inoculate fresh medium for optical density (OD) measurements in a plate reader or spectrophotometer over time (7) (Fig. 1A). Although there are exceptions when OD does not track viable cell number (8, 9), OD is widely used as a proxy for the density of cells in a culture (10, 11). While traversing such a growth curve, a cell population initially takes some time to accelerate in growth, experiences a period of rapid growth, and then decelerates as nutrients are consumed, waste products accumulate, or both (7). Although there can be substantial variability in the shape of a growth curve, many species qualitatively exhibit a sigmoidal shape that can be characterized by three parameters: (i) the maximal growth rate, *μ*_max_, which is the largest slope of the natural log of the OD over time, (ii) the lag time required to accelerate growth from stationary phase (*T*_lag_), and (iii) the maximum or final OD (*A*_max_); some cultures also exhibit a death phase (7). These parameters are typically extracted from a growth curve via fitting or direct calculation (12–21). Long-term evolution experiments (LTEEs) have demonstrated that all three can be under selection (4, 22), underscoring the importance of their accurate quantification. However, we have little understanding of how the technical aspects of model fitting and the methodological aspects of inoculation affect such quantifications.

The creation of genome-wide knockout libraries (23, 24) has motivated high-throughput measurements of population growth—a commonly used proxy for fitness—in microtiter plates, such as the systems biological characterization of essential gene knockdowns in Bacillus subtilis (5) and of Escherichia coli nonessential gene knockouts in liquid (25, 26) or embedded in a gel (26, 27). As the development of advanced genetic tools simplifies the creation of strain libraries (28–30), it is critical to ensure that OD measurements in multiwell plates provide reliable estimates of population-level growth parameters.

Here, we provide a detailed analysis of the requirements for achieving robust high-throughput measurements of fitness parameters. We quantified the importance of accurate background subtraction and established an assay of the sensitivity of the plate reader. We determined that measurements of maximal population growth rate and lag time are both sensitive to initial inoculation density, and we developed a mathematical model based on ordinary differential equations that accurately predicts this density dependence. We showed that completely removing residual glycerol from frozen stocks as a carbon source is critical for accurate measurements of E. coli growth in carbon-limited media, and we used such a strategy to reveal that evolution in a carbon-poor medium led to significant increases in the maximal growth rate of a population. We also established that the presence of glycerol during initial outgrowth from frozen stocks can have long-term impact on B. subtilis growth, causing long apparent lag times during subsequent culturing due to growth inhibition in a large majority of cells. Using transposon mutagenesis, we discovered that the increased lag time at least partially results from incorporation of glycerol during lipoteichoic acid synthesis. For large-scale high-throughput experiments, inoculating a large number of cultures from colonies is often too cumbersome; thus, these considerations are vital. Together, these findings provide a framework for accurate quantification of growth parameters and a roadmap for identifying and controlling for physiological factors that impact growth.

## RESULTS

### Accuracy of population density estimation from spectrophotometer absorbance readings is sensitive to the method of background subtraction.

To monitor maximum growth rate and lag time (defined here as time to reach half-maximum growth rate) during a bacterial population’s exit from stationary phase, it is standard practice to dilute a stationary-phase culture sufficiently that the spent medium transferred with the cells is a small fraction of the solution relative to the fresh medium. For a 100- to 1,000-fold dilution of a stationary-phase culture with an OD of ∼1 (typical for many species under high-nutrient conditions measured with our plate reader), the starting OD is ∼0.001 to 0.01. Thus, for species such as E. coli for which the maximum growth rate is achieved within 2 to 3 generations (31), the corresponding OD at the time of maximum growth rate can be low (≲0.01) (Fig. 1A), making it critical to ascertain whether OD can be accurately measured at low cell densities.

It is generally appreciated that correcting for the background absorbance improves the accuracy of growth measurements. However, the extent to which different background correction methods affect growth rate calculations has not been quantified. For a culture that is growing exponentially, the number of cells, N, grows over time as $N(t)=N_{0}2^{t/\tau }$, where $N_{0}$ is the number of cells at t=0, and τ is the doubling time. Thus, the growth rate of such a culture can be defined as the constant

(1) $$\frac{1}{N(t)}\frac{\mathrm{dN}(t)}{\mathrm{dt}}=\frac{\ln\;2}{\tau }$$

During outgrowth from stationary phase, when cells adapt their proteome to exploit the newly available nutrients (32), or after the cell density is sufficiently high that growth modifies the environment in a manner that impacts cellular physiology, the population does not grow exponentially. Nonetheless, analogous to exponential growth, we can define an instantaneous growth rate as

(2) $$g(t)=\frac{1}{N(t)}\frac{\mathrm{dN}(t)}{\mathrm{dt}}=\frac{d(\ln\;N(t))}{\mathrm{dt}}$$

Assuming that the OD measured by a plate reader is linearly related to N (OD=αN, where α is a scaling factor relating cell number to OD) and measurements are taken at time points t,t+Δt,t+2Δt, etc., the instantaneous growth rate at time t can be estimated as

(3) $$g(t)=\frac{1}{\mathrm{OD}(t)}\frac{d[\mathrm{OD}(t)]}{\mathrm{dt}}\approx \frac{1}{\mathrm{OD}(t)}\frac{\mathrm{OD}(t+\Delta t)-\mathrm{OD}(t)}{\Delta t}$$

To illustrate the importance of background subtraction, consider a culture in which ${\text{OD}}_{\text{raw}}\left(t\right)=\alpha N\left(t\right)+{\text{OD}}_{\text{bg}}$, where ${\text{OD}}_{\text{bg}}$ is the background absorbance in the absence of cells; ${\text{OD}}_{\text{bg}}$ is typically ∼0.1 (Fig. 1A). Without subtraction of ${\text{OD}}_{\text{bg}}$, the computed growth rate would be $\frac{1}{\alpha N(t)+{\text{OD}}_{\text{bg}}}\frac{\alpha N(t+\Delta t)-\alpha N(t)}{\Delta t}$ rather than $\frac{1}{\alpha N(t)}\frac{\alpha N(t+\Delta t)-\alpha N(t)}{\Delta t}$; the estimate of growth rate would be incorrect by a factor of $\frac{\alpha N(t)}{\alpha N(t)+{\text{OD}}_{\text{bg}}}$ (Fig. 1A). Thus, any estimate of growth rate without background (blank) subtraction is highly underestimated when in the regime ${\text{OD}}_{\text{bg}}≳\alpha N(t)$, which is likely given the time at which the maximum growth rate of many bacterial species is first achieved. For similar reasons, using the first time point of a growth curve as a proxy for the background absorbance leads to overestimation of the maximum growth rate, because the subtracted background is too large. Therefore, a method for correctly subtracting the background is crucial for accurate growth rate estimates.

We measured growth curves of E. coli MG1655 and estimated the instantaneous growth rate over time (see Materials and Methods), with and without blank subtraction. After subtracting the well blank, the maximal growth rate was 1.83 h^−1^ (doubling time of 22.7 min), which occurred at *t *= 1.52 h (Fig. 1B). Without blank subtraction, the maximal growth rate estimate was substantially lower (0.49 h^−1^), and the time at which this inaccurate estimate occurred was *t *= 3.0 h (Fig. 1B), illustrating the effects of omitting blank subtraction on lag time. To determine whether one empty well could be used as a general proxy for background absorbance, we quantified the absorbance of each well with medium before inoculating cells (see Materials and Methods). Blank values varied by ∼0.004 across the plate, while a single well’s blank value fluctuated by <0.001 over time (Fig. 1C). Blanking with a randomly selected well from the plate led to a wide variation in blanked growth curves (Fig. 1D) and maximum growth rates (Fig. 1E), with a standard deviation in growth rate estimate of 0.52 h^−1^ (Fig. 1F). Background subtraction with a well-specific blank led to substantially less variation in growth rate measurements, with a standard deviation in growth rate estimates for replicate cultures across the plate of 0.15 h^−1^ (Fig. 1F). Well-specific blanking also decreased the variability in lag measurements (see Fig. S1A in the supplemental material). The within-plate variability was sufficient to change the rank ordering of growth rates, which can lead to erroneous inferences. Thus, well-specific blanking is critical for accurate measurements of growth rate, because even comparisons in a single plate are confounded by within-plate variability.

10.1128/mBio.01378-20.1 FIG S1 Fitting parameters for a model of bacterial population growth that depends on inoculum size, and comparison of this model to experimental data. (A) Well-specific blank subtraction (teal) decreased the standard deviation in the estimate of lag time compared to that when subtracting a randomly selected blank in a 96-well plate (gray). (B) The instantaneous growth rate of E. coli MG1655 cells extracted from single-cell imaging on LB plus 1% agarose pads during emergence from stationary phase increased over the first hour. Shaded region indicates ±1 standard deviation. The black line is the Gompertz model with λ = 0.8 h^−1^ and δ = 0.5 h. (C) Simulated growth curves based on equations 4 to 6 in the main text captured, at least approximately, the decrease in lag time with increasing initial inoculum density. (D) Growth curves during a second passage in LB in which 2% glucose was added at various times. (E) Comparison of growth rate versus OD for the growth curves in panel D demonstrated that addition of glucose at 2 h or before led to growth-rate increase at later times when OD was >0.2, but glucose addition at 6 h or after had virtually no effect on growth. Download FIG S1, PDF file, 0.2 MB. Copyright © 2020 Atolia et al. 2020 Atolia et al. This content is distributed under the terms of the Creative Commons Attribution 4.0 International license.

### Sensitivity limit of the spectrophotometer also impacts the accuracy of growth rate measurements.

The ability to accurately measure changes in OD across a range of cell densities spanning several orders of magnitude is equally important for growth-rate calculations. Thus, we sought a general protocol for quantifying the limit of sensitivity and linear range of a given spectrophotometer. We inoculated serial dilutions of an overnight culture of E. coli MG1655 into fresh LB and measured the OD values. At low dilutions (OD > 0.63), the absorbance measured by the plate reader was not linearly related to cell density (Fig. 1G and H). Nonetheless, high OD measurements were able to be converted to cell-density estimates with a measured calibration curve (Fig. 1G), because the measurement coefficient of variation (CV; standard deviation/mean) remained very low (Fig. 1I). To determine the precision of plate reader measurements at high density, we converted the spread in growth curve replicates at the same dilution into standard deviations of cell density estimates and found that the range of accurate measurement was reliably extended up to the maximum tested expected OD of ∼3.5 (Fig. 1G), which is substantially higher than the typical corrected OD of ∼1 for a stationary-phase E. coli culture grown in LB.

For OD values less than ∼1, absorbances after well-specific blanking were linearly related to the dilution factor (Fig. 1G) and had a CV of <0.2 (Fig. 1I) down to a blank-corrected OD of ∼0.006, well below the plate background OD_bg_. For larger dilutions (OD < 0.006), the CV increased sharply (Fig. 1I), indicating that growth rate measurements in our spectrophotometer are likely to be very noisy for an OD of <0.006. We conclude that any growth curve should be initialized with an inoculum such that the maximum growth rate is achieved above a blank-corrected value of 0.006. This strategy is likely effective for calibrating and determining the sensitivity limit of any spectrophotometer.

### Maximum growth rates can depend strongly on initial inoculation density.

**FIG 2:**
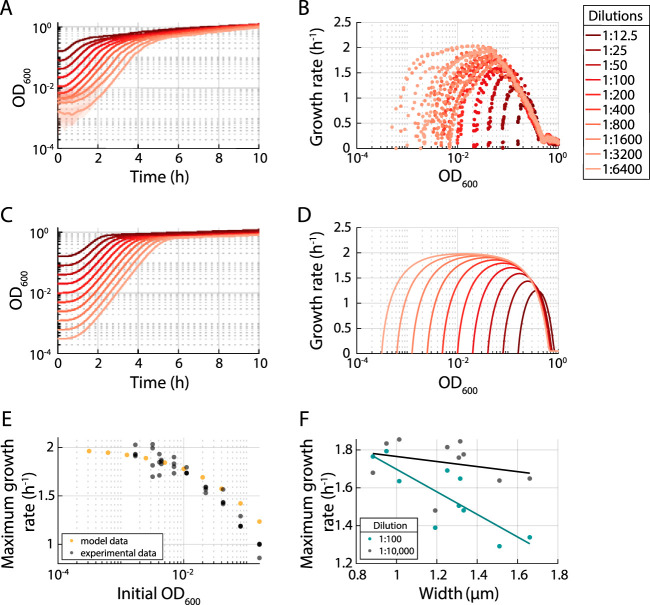
Growth rate is intrinsically linked to cell density due to nutrient depletion. (A) Growth curves of a dilution series from a single overnight culture of E. coli MG1655 displayed distinct growth behaviors, with slower initial growth for lower dilutions (higher initial cell density). (B) Instantaneous growth rates as a function of OD for the curves in panel B showed that lower dilutions resulted in lower maximum growth rates. Curves followed a common approximately linear downward trajectory after reaching their maximum growth rates, indicating that the entry to stationary phase is less affected by initial OD than lag time or maximum growth rate. (C) Nutrient depletion was sufficient to recapitulate experimental growth curves. Simulated growth curves for a model of growth based on nutrient depletion in equations 4 to 6 were similar to experimental data in panel A. (D) Nutrient depletion was sufficient to recapitulate the experimental relationship between OD and instantaneous growth rate. Instantaneous growth rate as a function of OD for the curves in panel C exhibited similar behaviors to the experimental data in panel B. (E) Nutrient depletion was sufficient to recapitulate the experimental relationship between initial inoculum size and maximum growth rate. The maximum growth rates of the experimental (B) and simulated growth curves (D) exhibited a quantitatively similar decrease with increasing initial inoculum density. (F) For a library of MreB mutants (34), the maximum growth rate computed from a growth curve initialized with a 1:100 dilution of an overnight culture (teal) decreased strongly with the mean cell width, while curves initialized from a 1:10,000 dilution (black) displayed higher, roughly constant maximum growth rates. The teal and black lines are least-squares linear fits to the data.

We previously found that the instantaneous population growth rate strongly correlated with OD across a library of B. subtilis mutants, despite their widely varied lag times, suggesting that cell density plays a major role in determining the population’s growth rate (5). Thus, to determine the optimal dilution for initializing growth curves, we systematically quantified how the inoculum cell density affects the trajectory of outgrowth from stationary phase. We diluted an overnight culture of E. coli MG1655 into fresh LB at ratios ranging from 1:12.5 to 1:6,400 in a 96-well plate and monitored the growth curves (Fig. 2A). To examine the relationship between OD and growth rate, we plotted each curve as growth rate versus OD (Fig. 2B). After correcting for the nonlinearity at high ODs (Fig. 1G) and subtracting the well-specific backgrounds, we found that the maximum growth rate achieved at low dilutions (e.g., 1:12.5) was lower than that at larger dilutions (Fig. 2B). As expected, each curve started at a different initial OD with a growth rate near 0 (Fig. 2B). Growth rate then increased and, for large dilutions (1:6,400), reached *μ*_max_ ≈ 2 h^−1^ (Fig. 2B). However, for lower dilutions, *μ*_max_ was substantially less than 2 h^−1^ and was attained at a higher OD (Fig. 2B). In each case, after reaching *μ*_max_, the growth rate declined approximately linearly as a function of log_10_(OD) along a common trajectory (Fig. 2B). Thus, before a population reaches its maximum growth rate, its growth rate trajectory is dependent on the initial cell density; thereafter, the growth curve follows a prescribed path independent of initial cell density.

To interrogate whether factors such as nutrient depletion or waste accumulation cause this density dependence, we developed a minimal model of population growth dynamics during passage in liquid culture. We assume that cell density *C* grows with an instantaneous growth rate *μ* dictated by the physiological status of the cells and the external environment:

(4) $$\frac{\mathrm{dC}}{\mathrm{dt}}=\mu C$$

Nutrients are consumed by growing cells at a rate proportional to their growth rate:

(5) $$\frac{\mathrm{dn}}{\mathrm{dt}}=\omega C-\beta \mu C$$

where n is nutrient concentration, ω represents the production of nutrients by the cells, and β is the nutrient consumption rate. We assume that the growth rate is a function of the nutrient concentration relative to a fixed nutrient concentration K (7); to model the transition from stationary phase into log phase, we assume that *μ* is related to the highest possible maximal growth rate μ* via a Gompertz relation (33):

(6) $$\mu =\mu *(\rho_{\max}e^{-e^{\frac{\lambda e(\delta -t)}{\rho_{\max}}+1}}+\rho_{\min})\frac{n}{n+K}$$

where $\rho_{\text{min}}$ is the lowest growth rate attained at high nutrient concentration normalized to μ*, $\rho_{\text{max}}=1-\rho_{\text{min}}$, λ governs how quickly *μ* increases, and δ is the time required to reach the maximum rate of growth rate change. We used single-cell growth data to obtain estimates of $\rho_{\text{max}}$, $\rho_{\text{min}}$, λ, and δ (Fig. S1B). We found that the simulated growth curves were relatively insensitive to the precise functional form of the acceleration in growth during lag phase.

We simulated growth curves based on equations 4 to 6, assuming that OD is proportional to C, with different initial densities C(t=0) and K=0.5 (where *n* = 1 is the maximum nutrient level), $\mu *=2\;{\text{h}}^{-1}$, β=0.8 h, $\rho_{\text{max}}=0.99$, $\rho_{\text{min}}=0.01$, $\lambda =0.8\;{\text{h}}^{-1}$, and δ=0.5 h. The kinetics of these growth curves (Fig. 2C) and the resulting relation between growth rate and OD (Fig. 2D) recapitulated our experimental findings reasonably well (Fig. 2B), including the roughly linear decrease in growth rate with OD after reaching $\mu_{\text{max}}$. Hence, nutrient depletion can largely explain the relations between $\mu_{\text{max}}$ and inoculation density (Fig. 2E), between lag and inoculation density (Fig. S1C), and more generally between growth rate and OD (Fig. 2D).

To experimentally distinguish between the effects of nutrient depletion and waste accumulation, we added 2% glucose to an E. coli culture at different times during growth in LB and monitored the effects on the growth curve (Fig. S1D). If nutrient depletion was the cause of growth rate slowdown, we surmised that the additional carbon would lead to a growth rate increase in early stationary phase. Early addition of the additional carbon before the culture saturated (≤2 h) allowed for maintenance of a higher growth rate between ODs of ∼0.2 to 0.5 (Fig. S1D and E), but later addition (after ≥6 h) had no effect on the growth curve relative to that for no glucose addition, due either to the build-up of waste that inhibits nutrient uptake or to the inability to switch to metabolizing glucose.

To illustrate the importance of these results, we examined the growth of a library of E. coli cell-size mutants (34). After a 1:10,000 dilution, all mutants exhibited similar maximum growth rates (Fig. 2F). However, after a 1:100 dilution, maximum growth rate was negatively correlated with the average cell width of each mutant (Fig. 2F) (34). This effect appears to reflect differences in outgrowth that prevented many of the mutants from attaining the higher maximum growth rate achievable at lower inoculation densities. These findings illustrate the importance of initiating growth curves with as low a cell density as possible without dropping below the plate reader’s limit of detection in order to avoid the region of decreasing maximum growth rate at high cell densities (Fig. 2F) that can distort comparisons between strains.

### Growth in a carbon-poor medium is highly sensitive to glycerol levels.

**FIG 3:**
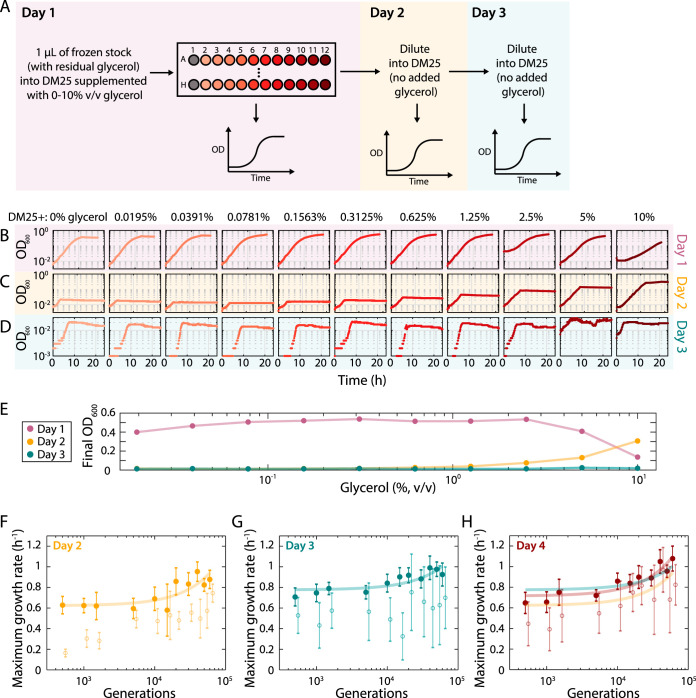
The presence of glycerol in carbon-poor medium increases the carrying capacity of that medium for E. coli. (A) Schematic of protocol to measure the effect of glycerol on growth. E. coli cultures were inoculated with 1 μl of a frozen stock in DM25 supplemented with various concentrations of glycerol. Growth was monitored over three passages, in which the latter two involved 1:200 dilutions into DM25 (without glycerol). (B) One microliter from a thawed frozen stock (20% glycerol) of E. coli REL606 was grown in minimal medium (DM25, 25 mg/l glucose) with various amounts of glycerol. On day 1, the carrying capacity was much higher than would be expected for the low glucose concentration (as seen with subsequent passages). (C) On day 2, the cultures were diluted into DM25, and the carrying capacity remained relatively high following the initially higher glycerol concentrations, presumably due to glycerol carryover. (D) By day 3, growth curves stabilized across all concentrations, and the increase in carrying capacity due to glycerol carryover no longer occurred. Note that the *y*-axis scale is different from those in panels B and C. (E) Dependence of final OD on glycerol concentration present during growth during the first passage. Final OD was much higher on day 1 than on day 2 and 3 for all concentrations other than 10% glycerol. On day 2, final OD increased with the glycerol concentration from the previous passage. On day 3, the dependence on glycerol concentration was gone, and the final OD was very low, as expected given the carbon-poor medium. (F to H) Outgrowth for multiple days minimized glycerol carryover and enabled accurate measurement of growth rate increases at low densities in the LTEE medium. Maximum growth rates computed using well-specific blanking (filled circles) in DM25 for the Ara-1 evolved line (40) were lower and noisier during the second growth passage (F) than during the third (G) and fourth (H) passages. Measurements in panels F and G were similar and revealed increases in maximum growth rate over the course of the LTEE. The lines are linear least-squares fits to the data on days 2 (yellow), 3 (teal), and 4 (maroon). Blanking based on a randomly selected well (F to H, open circles) led to generally lower growth-rate estimates and noisier trends; these data were slightly right-shifted to avoid overlap.

High-throughput methods often involve inoculation directly from a frozen stock rather than passaging through colonies, which could result in the transfer of variable amounts of glycerol, a cryoprotectant that ameliorates cell death during storage at low temperature (35, 36). Thus, we sought to identify factors, such as glycerol, that affect the growth of cultures inoculated directly from frozen stocks and then passaged multiple times. We hypothesized that glycerol would have persistent effects on growth, particularly in nutrient-poor media, because it can be utilized as a carbon source. Glycerol use that substantially increases the number of cells during the first passage would then perturb growth in later passages by changing the subsequent inoculation density (Fig. 2A). Such conditions are particularly relevant for strains generated by evolution experiments, which are often carried out in media with low carbon concentrations (37). To test the effect of glycerol on growth, we measured growth curves across a range of glycerol concentrations (Fig. 3A to D). We inoculated 1 μl from a −80°C freezer stock (previously grown in Davis minimal medium with 25 mg/l glucose [DM25]) of E. coli REL606 (38), the ancestral strain for the multidecade long-term evolution experiment (LTEE) carried out by Lenski and colleagues, into the evolution medium (DM25) supplemented with 0% to 10% (vol/vol) glycerol (Fig. 3A and B). The addition of glycerol mimics various levels of glycerol carryover from frozen stocks during inoculation. (However, we emphasize that the growth rate and competitive fitness assays performed by Lenski and collaborators prevents carryover of glycerol through repeated culturing in DM25, which acclimates the bacteria to the medium and other conditions of the LTEE prior to the fitness assays [39]). When we diluted these cultures 1:200, the resulting initial ODs substantially differed across glycerol concentrations, resulting in different growth kinetics with higher final OD values for cultures coming from higher glycerol concentrations on day 2 (Fig. 3C and E). After passaging the cultures a third time, the growth curves for the various glycerol concentrations were now quantitatively similar (Fig. 3D) with lower final ODs, as expected (Fig. 3E). Similar glycerol-dependent effects appeared when different amounts of a frozen stock were inoculated in DM25 (Fig. S2A and B). Thus, accurate quantification of growth requires multiple passages to eliminate the effects of glycerol on growth.

**FIG 4:**
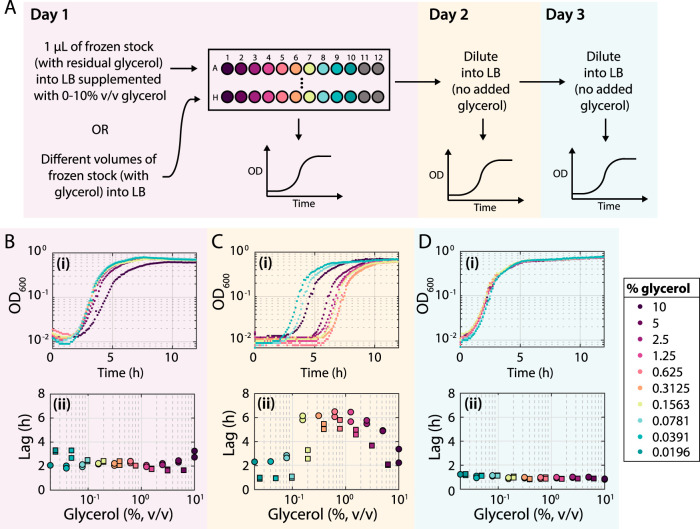
Growth with glycerol causes a large increase in lag time for B. subtilis in the subsequent passage. (A) Schematic of protocol to measure the effect of glycerol on growth. B. subtilis cultures were either inoculated in LB with various amounts of a frozen stock (see Fig. S3D and E in the supplemental material) or 1 μl of a frozen stock was used to inoculate LB supplemented with various concentrations of glycerol (as shown in B to D). Growth was monitored over three passages, in which the last two followed 1:200 dilutions into LB (without glycerol). (B to D) Growth curves on day 1 (Bi), day 2 (Ci), and day 3 (Di) of cultures inoculated with 1 μl of a frozen stock into LB supplemented with different concentrations of glycerol (in addition to the ∼0.075% transferred with the frozen stock). During the second passage (Ci), the cultures had similar maximum growth rates and carrying capacities, but intermediate inoculation amounts led to large increases in lag time. Growth curves were essentially identical during the third passage (Di). On day 1 (Bii), lag times were roughly constant for intermediate inoculation amounts (squares) or glycerol concentrations (circles), but lag times increased dramatically on day 2 (Cii); by day 3 (Dii) there was little difference in lag times across glycerol concentrations. These data indicate that glycerol caused the long-lag phenotype. Similar results were obtained when inoculating with different volumes of a frozen stock (Fig. S3).

10.1128/mBio.01378-20.2 FIG S2 Volume from a frozen stock used to inoculate a culture affects carrying capacity in a low-resource medium. (A) Various volumes of a frozen stock of E. coli REL606 were used to inoculate cultures grown in DM25. Dark to light red indicate three replicates of a small inoculum size (small but inexact volumes were picked from the frozen stock), while teal indicates an intentionally much larger inoculum volume. The large inoculum (teal) led to a higher final OD on the first day of growth. (B) When the cultures in panel A were diluted 1:200 into fresh DM25 and regrown for 24 h, the higher final ODs on day 1 translated into slightly higher ODs (teal) even on day 2. (C) E. coli REL606 cultures were inoculated by resuspending a small volume of the frozen stock in 200 μl of DM25, spinning down for 5 min at 8,000 × *g*, removing the supernatant, and resuspending the pellet in 200 μl of DM25 for growth. The curves represent four technical replicates. (D) When the cultures in panel C were diluted 1:200 into fresh DM25 and regrown for 24 h, the replicates behaved similarly and had a slightly lower final OD than the large inoculum in panel B (teal). (E) The E. coli Ara-1 line evolved a higher maximum growth rate over the course of 60,000 generations. One microliter of a frozen stock (20% glycerol) of each clone (shown by generation) sampled from the E. coli Ara-1 line (40) was grown in DM25 across four 24-h passages. On day 1, the carrying capacity was much higher than was achieved in later days in the low-glucose DM25 medium. The populations were diluted 1:200 on subsequent days into DM25, and growth curves were monitored in a plate reader. The maximum growth rate extracted from the curves was more accurately measured on days 3 and 4, and it increases over evolutionary time (Fig. 4). All curves are shown after subtracting the blank value of the corresponding well. Download FIG S2, PDF file, 2.4 MB. Copyright © 2020 Atolia et al. 2020 Atolia et al. This content is distributed under the terms of the Creative Commons Attribution 4.0 International license.

Combined with our finding that growth rate can be accurately measured even at low OD values with well-specific background subtraction (Fig. 1F), we realized that we could use multiple passaging to measure growth parameters in a high-throughput manner for the LTEE strains, enabling us to determine whether they changed systematically over the course of the LTEE. We examined 12 strains sampled from the Ara-1 population through 60,000 generations (40, 41). We diluted each culture into fresh DM25 and measured their growth curves. We then rediluted these overnight cultures 1:200 in fresh DM25 and measured their growth curves for three more passages. All strains attained relatively high ODs in the first passage due to the residual glycerol (Fig. S2E). In the second passage, growth rates were higher for the strains from later generations, but the measurements were noisy due to variations in inoculum levels (Fig. 3E). By the third passage, the noise decreased substantially, allowing us to observe that the growth rate had gradually increased over the 60,000 generations (Fig. 3F). By the fourth passage, maximum growth rates of the evolved strains had converged on values close to those measured the previous day (Fig. 3G), ranging from ∼0.65 h^−1^ to ∼1.08 h^−1^ from the earliest to the latest sample. Notably, the low stationary-phase density in DM25 (Fig. S2E) meant that even small amounts of noise greatly affected the measurements of growth rate; hence, well-specific blanking instead of random-well blanking was critical (Fig. 3E to G).

These data demonstrate that the serial-transfer regime of the LTEE has favored higher maximum growth rate, as predicted from theory (6) and measured previously over the first 20,000 generations (42). That previous work, however, involved using a glucose concentration much higher than that in the LTEE in order to achieve OD values suitable for measuring growth rates. Our new measurements highlight the importance of accurate blanking for quantifying growth behaviors, especially at low OD values.

### Growth in glycerol greatly increases B. subtilis lag time during subsequent passaging.

In addition to impacting bacterial growth in carbon-limited media, we hypothesized that glycerol could have other, species-specific effects on growth. To mimic the variable amounts of frozen stock that might be used to inoculate a culture, while also avoiding confounding differences in initial cell density, we inoculated a 96-well plate with 1 μl of frozen stocks of either B. subtilis 168 or E. coli MG1655 in LB supplemented with 0.1% to 10% glycerol and measured growth curves (Fig. 4A). For both species, during the first passage (day 1), all cultures exhibited approximately the same carrying capacity (Fig. 4Bi and S3Ai) and similar lag times (Fig. 4Bii and S3Aii).

10.1128/mBio.01378-20.3 FIG S3 Glycerol has little effect on E. coli growth in nutrient-rich LB. (A to C) Growth curves of E. coli MG1655 in LB supplemented with various amounts of glycerol on day 1 (Ai), diluted 1:200 into LB on day 2 (Bi), and diluted again 1:200 into LB on day 3 (Ci). There were slight glycerol-dependent effects on day 1 (Ai), but growth curves on days 2 (Bi) and 3 (Ci) were nearly identical for all cultures. Estimates of lag time from the growth curves are shown in Aii, Bii, and Cii. Lag times on day 1 (Aii) were longer than on days 2 (Bii) and 3 (Cii), which were quantitatively similar across glycerol concentrations. (D to F) Growth curves on day 1 (Di), day 2 (Ei), and day 3 (Fi) of cultures inoculated with various volumes of a frozen stock. During the second passage (Ei), the cultures had similar maximum growth rates and carrying capacities, but intermediate inoculation amounts led to much longer lag times. Growth curves were essentially identical during the third passage (Fi). Estimates of lag time from the growth curves are shown in Dii, Eii, and Fii. On day 1 (Dii), lag times were roughly constant for intermediate inoculation amounts of glycerol, but they increased dramatically on day 2 (Eii), before becoming uniform and independent of glycerol concentration on day 3 (Fii). These results are consistent with the increase in lag shown in Fig. 4C. (G to I) Growth curves of E. coli MG1655 in LB supplemented with various volumes of a frozen stock on day 1 (Gi), diluted 1:200 into LB on day 2 (Hi), and diluted again 1:200 into LB on day 3 (Ii). Inoculum volume affected initial OD, maximum growth rate, and lag time on day 1 (Gi), but growth curves on day 2 (Hi) and day 3 (Ii) were nearly identical for all cultures. Estimates of lag time from the growth curves are shown in Gii, Hii, and Iii. Lag times on days 2 (Hii) and 3 (Iii) were quantitatively similar across glycerol concentrations. Download FIG S3, PDF file, 1.4 MB. Copyright © 2020 Atolia et al. 2020 Atolia et al. This content is distributed under the terms of the Creative Commons Attribution 4.0 International license.

At high levels, glycerol causes catabolite repression and supports growth rates similarly to glucose (43). Hence, we speculated that glycerol could alter the physiological state of cells as they progress through another passage. To determine whether subsequent growth was affected by the prior presence of glycerol, we diluted all cultures 1:200 into fresh LB (without adding glycerol) and monitored growth in a plate reader for a second passage (day 2) (Fig. 4Ci and S3Bi). For intermediate concentrations between 0.16% and 5%, the lag time of B. subtilis cultures during this second passage increased to 6 h (Fig. 4Cii). This increased lag was specific to B. subtilis (Fig. S3Bii, Eii, and Hii), and went away during a third passage (Fig. 4D). Similar behavior was seen when inoculating a 96-well plate with fresh LB with various volumes (0.1 to 100 μl) of thawed frozen stocks of B. subtilis 168 (Fig. 4A and S3D to F). These data indicate that the second passage after revival of B. subtilis from a frozen stock is very sensitive to the glycerol concentration during initial inoculation.

To confirm that cellular responses to freezing were not required for the increase in lag time, we used 1 μl of an overnight culture grown from a frozen stock to inoculate LB supplemented with 0.1% to 10% glycerol; these cultures were now removed from freezing by a 24-h passage (Fig. S4A, purple). During the passage after glycerol addition, cells exhibited the expected increases in lag time (Fig. S4A, purple). Furthermore, when we inoculated the initial culture from a colony (instead of a frozen stock) into LB supplemented with glycerol, we saw similar increases in lag time during the subsequent passage (Fig. S4A, yellow) as from inoculating in various amounts of glycerol (Fig. 4C; Fig. S4A, teal) or different amounts of a frozen stock (Fig. S3E). These findings suggest that regrowth from a frozen stock displays little to no sign of cell death. The long-lag phenotype in the presence of glycerol for B. subtilis is distinct from the effects we observed in E. coli (Fig. 3B to E), in which the glycerol was metabolized and thus led to higher carrying capacities. Altogether, these data show that growth of B. subtilis 168 in glycerol can cause dramatic lag time increases during the subsequent passage for intermediate concentrations of glycerol. They highlight the importance of controlling for glycerol levels during high-throughput growth assays, which can be readily achieved by performing an additional passage.

10.1128/mBio.01378-20.4 FIG S4 Glycerol causes the long-lag phenotype in B. subtilis. (A) B. subtilis 168 was inoculated from a liquid culture (teal) or a colony (yellow) and grown in LB supplemented with various concentrations of glycerol. In both cases, there were similar lag increases on day 2 after 1:200 dilution and growth in LB. In contrast, there was no long-lag phenotype when cultures (initially inoculated with 1 μl of a frozen stock on day 1) were grown with glycerol on day 2 rather than day 1 (purple). (B) During the next dilution into LB (day 3), the cultures in panel A inoculated with liquid (teal) or colonies (yellow) exhibited short lags, while the glycerol cultures on day 2 (purple) displayed long lags, as expected due to growth in glycerol on the previous day. Growth of B. subtilis 168 in LB supplemented with glycerol for two consecutive days caused long lags at intermediate glycerol concentrations on day 2 with glycerol (C, gray) and day 3 without glycerol (D, gray), similarly to that with 1 day of growth in glycerol (maroon). Download FIG S4, PDF file, 0.1 MB. Copyright © 2020 Atolia et al. 2020 Atolia et al. This content is distributed under the terms of the Creative Commons Attribution 4.0 International license.

### Glycerol has dramatic and varied effects on B. subtilis single-cell growth.

**FIG 5:**
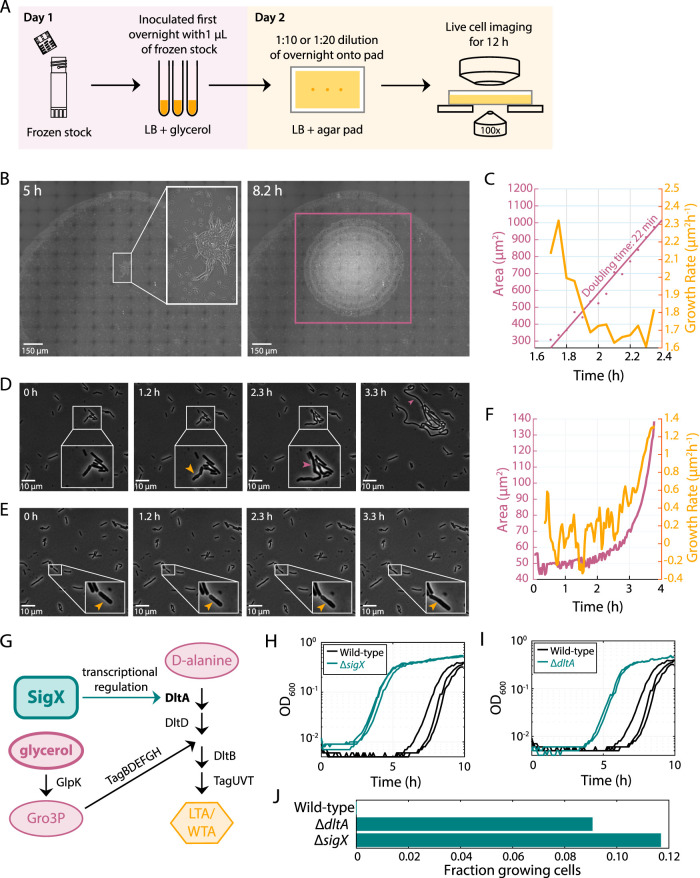
Growth of B. subtilis in intermediate glycerol concentrations results in highly heterogenous single-cell phenotypes during the subsequent passage. (A) Schematic of protocol for imaging single-cell growth during the passage after growth in glycerol. (B) Time-lapse images of a stitched set of 144 fields of view covering the entire spot of cells on an LB agar pad after a passage in LB plus 0.3125% glycerol. Inset, enlarged view of the only cell that exhibited growth across the entire spot. The purple box at right highlights the resulting microcolony at 8.2 h. (C) Quantification of cell area (purple) and instantaneous growth rate (yellow) of the sole growing cell in panel B revealed a maximum growth rate corresponding to a doubling time of 22 min. (D) Time-lapse images of cell growth on an LB agar pad after growth in liquid LB plus 0.625% glycerol. The cell (inset, enlarged) started growing immediately, and bulged (yellow arrow) and filamented for ∼3 h (purple arrow). (E) Time-lapse images of cell growth (yellow arrow; inset, enlarged) on an LB agar pad after growth in liquid LB plus 0.625% glycerol; this cell did not start growing until after ∼2 h. (F) Quantification of cell area (purple) and instantaneous growth rate (yellow) of the cell in panel E, which had a longer lag and lower growth rate than the cell in panel C. (G) Schematic of regulatory pathway for biosynthesis of LTA and WTA involving SigX and DltA. SigX transcriptionally regulates *dltA* and 47 other genes, some of which are involved in biosynthesis of teichoic acids. Glycerol is converted to *sn*-glycerol 3-phosphate (Gro3P); Gro3P, in conjunction with an intermediate product made by DltA, is necessary for the production of teichoic acids. (H, I) Deletions (teal) of *sigX* (H) and *dltA* (I) resulted in a shorter lag than the wild type (black). (J) During time-lapse imaging of stationary-phase outgrowth on an LB agar pad after one passage in LB plus 0.3125% glycerol, 0% (0/701), 9% (132/1458), and 12% (148/1267) of wild-type, Δ*dltA*, and Δ*sigX* cells, respectively, exhibited any growth after 5 h. The larger fraction of Δ*dltA* and Δ*sigX* cells relative to wild-type cells that exhibited growth is consistent with the shorter lag time in bulk liquid culture of these strains (H and I).

We were surprised by the increased lag times in B. subtilis growth after passaging with intermediate glycerol concentrations and sought to investigate the cell physiology underlying this phenomenon. We used time-lapse microscopy to monitor the growth of cells on LB agar pads following growth in liquid LB at the glycerol concentrations associated with the longest population lag times (Fig. 4C). For cells grown in liquid LB with 0.3125% glycerol (∼6-h lag time) (Fig. 4Cii), we imaged an entire ∼2 mm-diameter spot by capturing a grid of 144 overlapping fields of view (Fig. 5A). Of the ∼10^4^ cells in one spot, only a single cell grew, and its descendants took over the entire spot over 12 h of imaging (Fig. 5B). That cell exhibited growth as soon as imaging began, and after 1.7 h, its lineage exhibited a doubling time of ∼20 min (Fig. 5C), indicating the extreme heterogeneity in this population. In other spots (*n *= 2), we observed no growth of any cells. Such extreme bottlenecks should be avoided for most applications.

For cells grown in LB with 0.625% glycerol (∼6-h lag time) (Fig. 4Cii), we again observed a small fraction of growing cells (<3%) (Fig. 5D). The cells that grew showed multiple phenotypes. Some cells started growing immediately (Movie S1); some initially bulged along the cell body and then filamented for ∼2.5 h before the first division (Fig. 5D). Other cells did not grow for >2 h (Fig. 5E) and thereafter grew more slowly than normal (Fig. 5F; see also Movie S2). A third subset periodically shrank and had a high death rate (Movie S3). For one cell, no growth was observed for the first 3 h; afterwards it engaged in short phases of growth and shrinking, with small blebs forming during growth (Fig. S5A). It eventually divided, and many of its progeny also exhibited periodic shrinking (Movie S3) and death through explosive lysis (Fig. S5A). Thus, growth in glycerol clearly disrupts cell shape as well as growth out of stationary phase in multiple ways.

10.1128/mBio.01378-20.5 FIG S5 Mutations in genes involved in teichoic acid production shorten glycerol-induced lag times in B. subtilis. (A) Time-lapse images of a cell on an LB agar pad after growth in liquid LB plus 0.625% glycerol; this cell did not grow for >3 h and then engaged in short bursts of growth and shrinking (yellow arrows). Small blebs formed on another cell during growth (white arrows), and many of its progeny also exhibited periodic shrinking and died by explosive lysis (purple arrows). (B) A transposon mutagenesis screen yielded two positive hits (yellow and purple) of libraries with shorter lag times during passaging after growth in LB plus 1.25% glycerol than the parental wild-type strain (black). Light-purple and light-yellow curves show 6 biological replicates. (C) Single colonies isolated from the two libraries in panel A had shorter lag times during passaging after growth in LB plus 1.25% glycerol than the parental wild-type strain (black). Sequencing revealed that the colonies have insertions in *dltA* (teal) and *sigX* (maroon), both of which are linked to lipoteichoic acid synthesis. Light teal and light maroon curves show 5 biological replicates. Reintroduction of the transposon insertions (teal) in *dltA* (D) and *sigX* (E) from the positive hits of the screen in panel C resulted in a shorter lag times than the wild type (black). Two independent colonies were tested for each insertion. (F) Time-lapse imaging of Δ*dltA* and Δ*sigX* cells on LB agar pads during emergence from stationary phase after one passage in LB plus 0.3125% glycerol. Arrowheads denote examples of cells with aberrant morphologies. Download FIG S5, JPG file, 2.7 MB. Copyright © 2020 Atolia et al. 2020 Atolia et al. This content is distributed under the terms of the Creative Commons Attribution 4.0 International license.

10.1128/mBio.01378-20.7 MOVIE S1 A small fraction of cells exhibited bulging and filamentous growth after overnight passage in LB plus 0.625% glycerol, related to Fig. 5D. Wild-type B. subtilis 168 cells from an 18-h culture in LB plus 0.625% glycerol were spotted onto an LB-agarose pad and imaged every 2 min at 37°C. This experiment was done in tandem with those shown in Movies S2 and S3. Download Movie S1, MOV file, 2.6 MB. Copyright © 2020 Atolia et al. 2020 Atolia et al. This content is distributed under the terms of the Creative Commons Attribution 4.0 International license.

10.1128/mBio.01378-20.8 MOVIE S2 A small fraction of cells exhibited long lags after overnight passage in LB plus 0.625% glycerol, related to Fig. 5E. Wild-type B. subtilis 168 cells from an 18-h culture in LB plus 0.625% glycerol were spotted onto an LB agar pad and imaged every 2 min at 37°C. This experiment was done in tandem with those shown in Movies S1 and S3. Download Movie S2, MOV file, 2.6 MB. Copyright © 2020 Atolia et al. 2020 Atolia et al. This content is distributed under the terms of the Creative Commons Attribution 4.0 International license.

10.1128/mBio.01378-20.9 MOVIE S3 Some cells exhibited repeated phases of growth and shrinking during regrowth after overnight passage in LB plus 0.625% glycerol, related to Fig. S5A. Wild-type B. subtilis 168 cells from an 18-h culture in LB plus 0.625% glycerol were spotted onto an LB agar pad and imaged every 2 min at 37°C. For one cell, no growth was observed for the first 3 h; afterward, it engaged in short phases of growth and shrinking, with small blebs forming during growth. Some of its progeny also exhibited periodic shrinking and death through explosive lysis. This experiment was done in tandem with those shown in Movies S1 and S2. Download Movie S3, AVI file, 16.7 MB. Copyright © 2020 Atolia et al. 2020 Atolia et al. This content is distributed under the terms of the Creative Commons Attribution 4.0 International license.

### A screen links glycerol-induced lag to genes involved in teichoic acid synthesis.

The discovery of B. subtilis’s long lag and consequent fitness defect induced by intermediate glycerol concentrations (Fig. 4) presented the opportunity to identify genetic determinants of this phenotype, as mutants with a shorter lag would be enriched in the population. To gain insight into the underlying mechanism, we carried out an unbiased genetic screen by creating six independent pooled libraries of transposon mutants (see Materials and Methods) (44) in the wild-type strain. We grew the libraries in LB plus 1.25% glycerol, a concentration that induced a long lag time in the wild type (Fig. 5Eii). Further passaging of the libraries once in LB plus 1.25% glycerol and once more in LB revealed two libraries that evolved shorter lag times (Fig. S5B). We isolated single colonies and verified that they had a similar phenotype to the library from which they were isolated (Fig. S5C). Sequencing their transposon insertion sites identified two mutations: in *sigX*, which encodes a sigma factor that regulates modification of the cell envelope and resistance to cationic antimicrobial peptides (45), and in the start codon of *dltA*, which encodes a d-alanine ligase required for modification of wall teichoic acids (WTA) and lipoteichoic acids (LTA) (46). Note that *dltA* is part of the *sigX* regulon (Fig. 5G) (45). We verified these hits by reintroducing each transposon insertion into the parental strain (Fig. S5D and E) and by deleting the gene (*sigX* or *dltA*) and then showing that these constructs exhibited the same reduction in lag (Fig. 5H and I). Time-lapse imaging revealed that Δ*sigX* and Δ*dltA* cultures still exhibited regrowth heterogeneity, but with a much larger fraction (∼10%) of growing cells than the wild type (Fig. 5J). During stationary-phase outgrowth, Δ*sigX* and Δ*dltA* cells exhibited aberrant morphologies (Fig. S5F) similar to those taken on by the few growing wild-type cells after passaging in LB plus 0.625% glycerol (Fig. 5D and E; Fig. S5A). The thick peptidoglycan cell wall of Gram-positive bacteria is intercalated with wall teichoic acids, which are covalently bound to peptidoglycan, and lipoteichoic acids, which are tethered to the membrane by a lipid anchor (47). Production of both wall teichoic acids and lipoteichoic acids requires substantial amounts of glycerol (48). Thus, it appears that teichoic acid production is linked to the glycerol-dependent long-lag phenotype in B. subtilis.

## DISCUSSION

As microbiology research has expanded and flourished, so has the appreciation of the sensitivity of microbial physiology and cellular structure to environmental conditions. Uncovering the details of these sensitivities will be critical to quantitative understanding of growth behaviors across microbial strains and species, as will establishing the resolution and robustness of the equipment used to measure growth. We have described a general strategy for measuring an instrument’s limit of detection and range of linearity and demonstrated that, with proper protocols (see Materials and Methods), a wide range of growth behaviors can be accurately quantified.

The dependence of maximum growth rate on initial cell density presents complications similar to the antibiotic inoculum effect, whereby the sensitivity to certain drugs increases at lower cell density (49). Comparisons of growth rate and lag time between strains would ideally employ similar initial cell concentrations. However, fulfilling such a condition can be challenging due to strain differences in cell shape (50), yield in a given medium, and cell survival in and recovery from stationary phase. Moreover, some species may have growth dynamics and carrying capacities that are inoculum dependent. Given these complications, the acquisition of growth curves across a range of initial densities to map the range of growth behaviors would enhance the ability to compare strains and species. Our model based on ordinary differential equations is general and hence can be used for many microbes; in particular, it can help to correct for differences in growth rate due to variation in initial inoculum size. It is also important to note that waste accumulation should be mathematically equivalent to nutrient depletion if waste products inhibit the uptake of some nutrients, which means our model is even more broadly applicable. However, other factors may prove important for modeling growth curves, such as pH changes that are known to inhibit growth (51).

Although spectrophotometers that read microtiter plates are quite sensitive to small changes in OD (Fig. 1D and E), our analyses establish that it is critical to minimize noise that introduces complexities when calculating growth metrics; this noise minimization can be best achieved by blanking each individual well separately. This modification to protocols was relatively straightforward, and it positioned us to quantify the contribution of increased growth rate to the fitness gains observed in the LTEE with E. coli (Fig. 3). In particular, this method allowed us to show unequivocally that faster exponential growth had been selected even at the low glucose concentration and consequently low OD values of the LTEE; this conclusion was obscured without well-specific blanking (Fig. 3F to H). The ∼66% increase in maximum growth rate that we measured is reasonably close to the ∼70% increase in relative fitness obtained through competing late-generation samples against their ancestor (39). That relative fitness is calculated as the ratio of the realized growth rates of the evolved and ancestral bacteria over a full 24-h transfer cycle, including lag, growth, and stationary phases. Thus, other growth parameters can also affect relative fitness, including differences in lag time and carrying capacity (52), which complicates a direct comparison between maximal growth rate and relative fitness. In addition, cross-feeding interactions have evolved in some LTEE populations, and these interactions may affect the post-maximum growth rates of the competitors as cells exhaust the limiting glucose, consume by-products, and transition into stationary phase (53–55). In any case, our new protocol and growth rate measurements demonstrate the value of subtracting the blank of each well to minimize noise, especially at low OD values. These growth rate data also imply that utilization of glucose has become much faster over the LTEE, and more generally, it may be possible to evolve many bacterial species to grow at higher rates under specific nutrient conditions. Given the correlation between cell size and fitness in the LTEE (56), future studies might use these strains to explore whether the “Growth Law” that links nutrient-dependent growth rate and cell size (57) has changed over the course of evolution. In fact, it was previously shown that that the relation is not constant between the ancestor and an evolved strain isolated after just 2,000 generations (58).

The dramatic increases in lag time that we observed in B. subtilis (Fig. 4C) indicate that glycerol can have lasting physiological effects that impede the future growth of cells. We did not observe this lag phenotype in E. coli (Fig. S3B). This difference is consistent with the requirement of Gram-positive bacteria for glycerol to synthesize teichoic acids, which they incorporate into the cell envelope. Without multiple dilutions to mitigate the glycerol-induced long-lag phenotype, a severe bottleneck in which very few cells are responsible for outgrowth can occur (Fig. 5B), which may complicate interpretation of experimental results. The conventional approach to streaking single colonies before starting liquid cultures avoids this problem (Fig. S4A and B), likely due to the extreme dilution of the glycerol concentration. While streaking may be prohibitive for large strain collections or certain species and communities, washing the initial inoculum to remove the glycerol is also sufficient (Fig. S2C and D).

Our transposon mutagenesis screen linked the glycerol-induced long-lag phenotype in B. subtilis to the incorporation of glycerol into lipoteichoic acids (Fig. 5H and I). Our data also revealed several unusual physiological and morphological impacts of glycerol in the subsequent passage, including filamentation (Fig. 5D), bulging (Fig. 5E), lysis (Fig. S5A), and repeated cycles of growth and shrinkage (Fig. S5A). Some of these phenotypes are consistent with previous observations connecting lipoteichoic acid synthesis with cell division (59). Moreover, the arrest of growth in the vast majority of cells (Fig. 5E) indicates that their physiological state was sensitized by previous exposure to even small amounts of glycerol, such that growth remained inhibited even after glycerol was no longer present. This phenomenon connects teichoic acid synthesis to growth inhibition and lag phase for the first time, and it points to a severe bottleneck that might cause additional experimental complications. This surprising phenotype also highlights the potential for other physiological history-dependent effects on growth.

In the process of dissecting the seemingly simple process of measuring bacterial growth, we developed a refined protocol that enabled precise quantitative measurements. This precision, in turn, led to the discovery of new biological phenomena. The microbial world is stunningly diverse, and we currently know very little about the growth kinetics of the vast majority of microbes. Our work provides a powerful framework to analyze the growth characteristics of microbial species in high-throughput assays.

## MATERIALS AND METHODS

### Detailed protocol for growth curve measurements with well-specific blanking.

### (i) Optional.

Determine the limit of detection and linear range of the plate reader.

1. Pellet 10 ml of a saturated culture and separate the spent supernatant.
2. Resuspend the pellet in a small amount (∼1 ml) of spent supernatant to concentrate cells.
3. In a 96-well plate, add 200 μl of spent supernatant to all wells to measure well-specific blanks:
  a. Place a clear plastic plate seal with laser-cut or poked holes on the 96-well plate.
  b. Measure OD in the plate reader for 30 min every 3 to 7 min; this interval allows condensation on the plate seal to subside and the OD measurements to stabilize.
4. Remove enough medium from each well in the 96-well plate to allow for a 1.2-fold serial dilution of the concentrated culture from step 2 using spent supernatant until the OD of the final dilution is likely well below the limit of detection (∼10^−4^ should be sufficient).
5. Place a clear plastic plate seal with laser-cut or poked holes on the 96-well plate, taking care to avoid splashing that could cause contamination.
6. Measure OD for the 96-well plate for 30 min every 3 to 7 min; this interval allows condensation on the plate seal to subside and the OD measurements to stabilize.

### (ii) Passage 1.

Inoculate an overnight culture from a frozen stock and ensure that the culture has reached saturation. Since growth directly from the frozen stock should not be used for growth quantification, OD measurements are optional and well-specific blanking is not necessary.

1. Inoculate at least 200 μl of fresh medium with a small volume of a frozen stock (ideally <1 μl).
2. Use 1 μl of the dilution to inoculate 200 μl of fresh medium in each well of a 96-well plate. This second dilution reduces the carryover of components of the frozen stock such as glycerol.
3. Place a clear plastic plate seal with laser-cut or poked holes above each well on the 96-well plate.
4. (Optional) Measure OD over time in a plate reader for the time frame of interest (e.g., 18 to 24 h).

### (iii) Passage 2.

Dilute passage 1 cultures into fresh medium and quantify growth parameters. To ensure accurate quantification of maximum growth rate and lag time, well-specific blanking is required.

1. Fill each well in a 96-well plate with 200 μl of fresh medium.
2. Place a clear plastic plate seal with laser-cut or poked holes on the 96-well plate.
3. Measure OD in a plate reader for 30 min every 3 to 7 min; this interval allows condensation on the plate seal to subside and the OD measurements to stabilize.
4. Remove the plate seal, taking care to avoid splashing that could cause contamination.
5. Dilute passage 1 cultures into 200 μl of fresh medium in a 96-well plate. The dilution ratio should be selected so that the OD of the diluted culture is above the limit of detection of the plate reader; typically, 1:200 is reasonable.
6. Place a plastic plate seal with laser-cut or poked holes onto the 96-well plate, taking care to avoid contamination, as in step 4. The same seal that was removed in step 4 can be reused, as long as it is placed in the same orientation as before.
7. Measure OD over time in a plate reader for the time of interest.

### (iv) Passage 3.

Dilute passage 2 cultures into fresh medium and quantify growth parameters. To ensure accurate quantification of maximum growth rate and lag time, well-specific blanking is required. These data are used to determine if the growth curves have stabilized and whether there are any unintended factors remaining from the frozen stock that affect outgrowth.

1. Repeat steps from passage 2.

### (v) (Optional) Passage 4.

Dilute passage 3 cultures into fresh medium and quantify growth parameters. To ensure accurate quantification of maximum growth rate and lag time, well-specific blanking is required. These data are only necessary if the growth curves in passage 3 did not stabilize relative to those in passage 2.

1. Repeat steps from passage 2.

### Strains and media.

Table S1 in the supplemental material lists the strains and their genotypes used in this study. Strains were grown in LB (lysogeny broth with 10 g/l tryptone, 5 g/l NaCl, and 5 g/l yeast extract) or DM25 (Davis minimal broth [60] supplemented with 2 mg/l thiamine hydrochloride and 25 mg/l glucose [37]). Both media were supplemented with 0% to 10 % (vol/vol) glycerol where indicated. When stated, antibiotics were used as follows, unless indicated otherwise: kanamycin (5 μg/ml) and MLS (a combination of 0.5 μg/ml erythromycin and 12.5 μg/ml lincomycin).

10.1128/mBio.01378-20.10 TABLE S1 List of strains used in this study. Download Table S1, DOCX file, 0.1 MB. Copyright © 2020 Atolia et al. 2020 Atolia et al. This content is distributed under the terms of the Creative Commons Attribution 4.0 International license.

### Bacterial growth.

For all glycerol experiments, to avoid potential issues associated with accumulated damage over time spent in the freezer, we first made new frozen stocks of E. coli MG1655 and B. subtilis 168 by growing a single colony overnight in LB and then rapidly freezing a 1:1 mixture of the culture with 50% glycerol in a −80°C freezer. All strains (Table S1) were inoculated from −80°C freezer stocks into 200 μl of the medium of interest in shallow 96-well plates (Greiner) and grown overnight for 18 h while shaking at 37°C. Overnight cultures were diluted 1:200 in fresh medium for plate reader experiments and either 1:10 or 1:20 into fresh medium for microscopy.

### Plate reader absorbance measurements.

A shallow 96-well plate was filled with fresh medium, and a plate seal (Excel Scientific) with laser-cut holes was placed on top. The background absorbance at 600 nm (OD_600_) was measured for 30 min to allow the readings to stabilize. Overnight cultures were diluted 1:200 into this plate using the same plate seal and grown with shaking at 37°C in a BioTek Epoch 2 plate reader for 18 to 24 h. OD_600_ was measured at 7.5-min intervals. For the resulting growth curves, the slope over a sliding window (for smoothing) was computed to determine the instantaneous growth rate, and from that, the lag time (defined as the time to half-maximal growth rate) was calculated. Additionally, the natural logarithm of OD_600_ was fit to the Gompertz equation (12) to quantify lag time and maximal growth rate. The two methods for calculating lag times and maximum growth rates were highly correlated (Fig. S6) for growth curves of E. coli. The first method was more appropriate for growth curves that did not reach saturation or exhibited a distinct shape (e.g., due to a diauxic shift). Thus, we used the first method to quantify lag time and maximal growth rate, except as noted.

10.1128/mBio.01378-20.6 FIG S6 Fitting a Gompertz function and a linear model to a sliding window of log-transformed OD values provides nearly identical estimates of growth parameters in E. coli. (A) Maximum growth rates calculated using a Gompertz fit or using a sliding window were highly correlated (*x* = *y* line in yellow). (B) Lag time calculated using a Gompertz fit was highly correlated (*x* = *y* line in teal) with the time to reach half-maximum growth rate calculated using a sliding window fit. Download FIG S6, PDF file, 0.3 MB. Copyright © 2020 Atolia et al. 2020 Atolia et al. This content is distributed under the terms of the Creative Commons Attribution 4.0 International license.

### Microscopy.

Cells were diluted 1:10 or 1:20 (depending on the overnight culture OD) into fresh LB. For time-lapse imaging, 1 μl of cell culture was placed onto a large pad (the size of a 96-well plate) composed of 1.5% agar plus LB. Such a large pad was used to avoid oxygen depletion in the pad over the course of imaging, because B. subtilis cells lyse under oxygen-limited conditions (8). The cells were imaged using a Nikon Eclipse Ti-E inverted fluorescence microscope with a 100X (numerical aperture [NA] 1.40) oil immersion lens objective and integrated using μManager v. 1.4 (61). Cells were maintained at 37°C during imaging with an active-control environmental chamber (HaisonTech).

### Strain construction.

We constructed strains to study the role of LTA synthesis in glycerol-induced lag using SPP1 phage transduction (62). The donor strain was grown for >6 h in TY medium (LB supplemented with 0.01 M MgSO_4_ and 0.1 mM MnSO_4_ after autoclaving). Ten-fold dilutions of SPP1 phage were added to the culture, and 3 ml of TY soft (0.5%) agar were then mixed with the cell-phage mixture and poured over a TY plate (1.5% agar) before overnight incubation. We chose a plate that exhibited nearly complete clearing of cells and thus without many phage-resistant mutants. Five milliliters of TY medium were added to this plate, and a 1-ml filter tip was used to scrape up the soft agar. This soft agar-liquid mix was filtered through a 0.4-μm filter (Fisher Scientific). The phage were added to a stationary-phase culture (grown for 6 to 10 h) of the recipient strain (10 μl undiluted phage plus 100 μl recipient cells and, optionally, 900 μl TY medium) in TY medium, incubated at 30°C for 30 min, and then plated onto LB plus antibiotic and 0.01 M sodium citrate (sodium citrate was omitted for MLS selection). Plates were incubated for 24 h at 30°C, and transductants were streaked for single colonies to eliminate the phage.

The back-crossed transposon mutagenesis strains and deletion strains (Δ*sigX* and Δ*dltA*) were constructed via double-crossover integration by transformation of genomic DNA using standard transformation procedures (63, 64). We performed genomic DNA extractions of the original transposon mutagenesis strain, BKK23100 (*sigX*), and BKK38500 (*dltA*) using the Promega Wizard kit.

### *mariner* transposon library construction.

The *mariner* transposon was transduced into the parent strain as described above. The *mariner* plasmid has a temperature-sensitive origin of replication, a transposase, and a transposon that is marked with kanamycin resistance; the backbone plasmid is marked with MLS resistance (65). Before transduction of the *mariner* transposon, the parent strain (CAG74168) was streaked for single colonies on MLS plates at 30°C. One colony for each library was grown overnight in 3 ml LB plus kanamycin in a roller drum at room temperature. Transposon-insertion libraries were created by plating 10-fold dilutions of the cultures on LB plus kanamycin plates pre-warmed to 37°C and incubating them overnight at 37°C. The dilution that contained very dense colonies was scraped to create the library, and the higher dilutions were used to estimate the number of transposon mutants per library.

### Transposon library screen.

The transposon library was screened by using the entire library to inoculate a 5 ml LB plus 1.25% glycerol culture. This culture was diluted 1:200 after 18 h of growth into 200 μl of LB plus 1.25% glycerol, grown for 18 h with shaking at 37°C, and diluted 1:200 into LB. After two passages of growth in LB plus glycerol and one passage in LB, the libraries with a shorter lag time were streaked for single colonies on LB plates. Five single colonies were picked for each positive hit and used to inoculate a 200-μl LB culture. After 18 h, the culture was diluted 1:200 into LB plus 1.25% glycerol. The culture was grown for 18 h and diluted 1:200 into LB. After three passages, the positive hits were grown in 5 ml of LB for genomic DNA extraction.

### Mapping transposon insertions using inverse PCR.

Genomic DNA was extracted using the Promega Wizard Genomic DNA purification kit, digested with Sau3AI at 37°C for 90 min, and then heat-inactivated at 65°C for 20 min. The reaction mixture contained 15.5 μl Milli-Q H_2_O, 2 μl NEBuffer1.1, 2 μl digested genomic DNA, 2 μl 10X bovine serum albumin, and 0.5 μl Sau3AI. The digested DNA was ligated using T4 ligase at room temperature for at least 1 h; the reaction mixture contained 2 μl T4 DNA ligase buffer, 15.5 μl Milli-Q H_2_O, 2 μl digested DNA, and 0.5 μl T4 DNA ligase. Inverse PCR was carried out with the ligated DNA using Phusion polymerase with the primers IPCR1 (5′-GCTTGTAAATTCTATCATAATTG-3′) and IPCR2 (5′-AGGGAATCATTTGAAGGTTGG-3′). Each reaction contained 33 μl Milli-Q H_2_O, 10 μl 5X HF buffer, 2 μl ligated DNA, 2 μl IPRC1, 2 μl IPCR2, 1 μl 10 mM deoxynucleoside triphosphates (dNTPs), and 0.2 μl Phusion polymerase. The PCR program was as follows: 98°C for 30 s, 30 cycles of 98°C for 10 s, 58°C for 30 s, and 72°C for 60 s, 72°C for 10 min, and hold at 4°C. PCR products were gel-purified and sequenced using the IPCR2 primer. The sequences were mapped onto the B. subtilis 168 genome using BLASTN.

### Data availability.

All data used in the manuscript are growth curves, time-lapse microscopy images, or transposon sequencing. All data are available upon request from the corresponding author.
