# Supplementary figures and images for: Environmental and Physiological Factors Affecting High-Throughput Measurements of Bacterial Growth
Source: mBio. 2020 Oct 20;11(5):e01378-20. doi: 10.1128/mBio.01378-20 (PMC7587430; doi:10.1128/mBio.01378-20)

**A**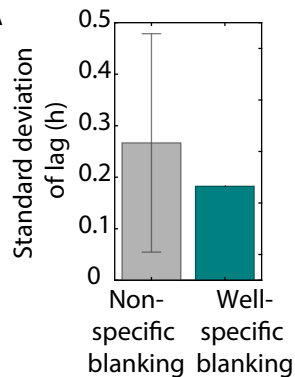**B**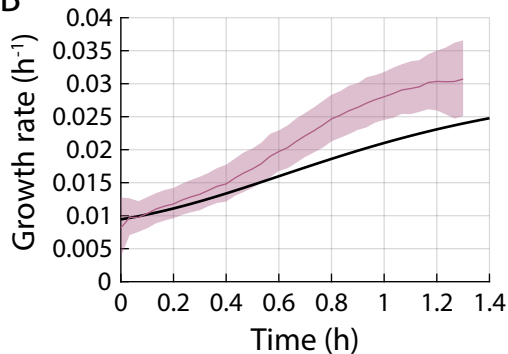**C**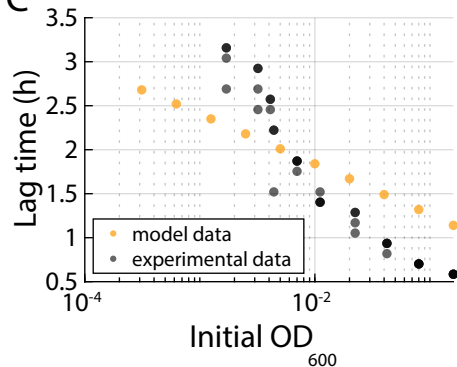**D**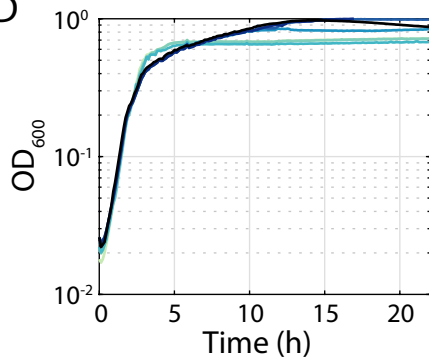**E**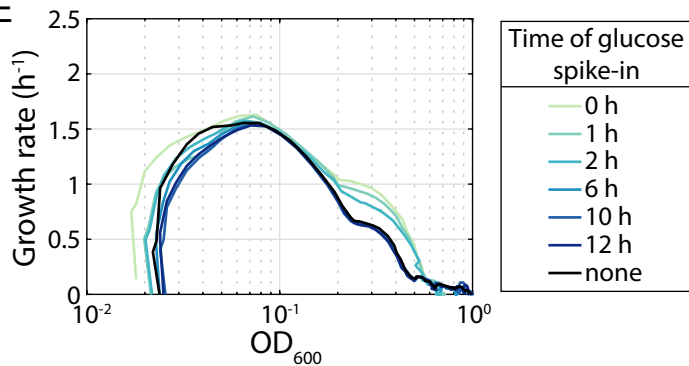

Supplement: FIG S1 [file mBio.01378-20-sf001.pdf]

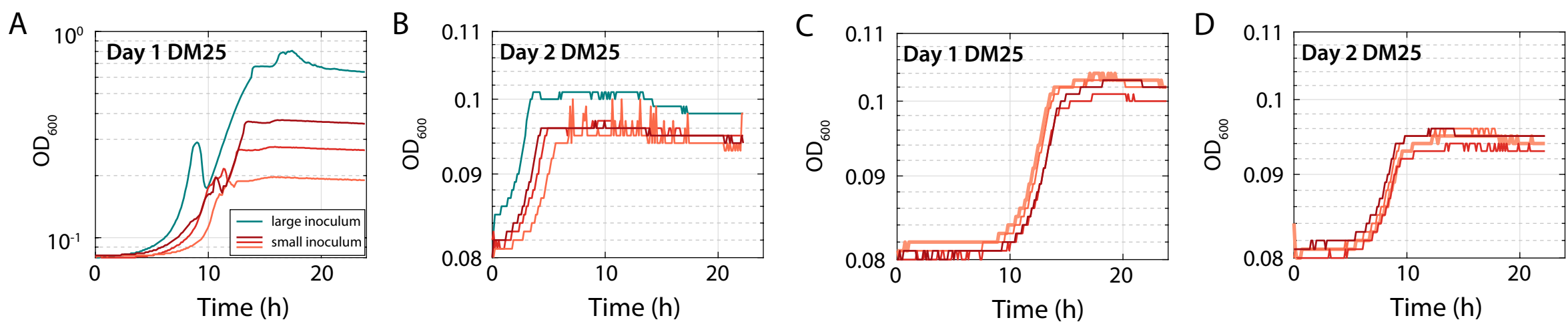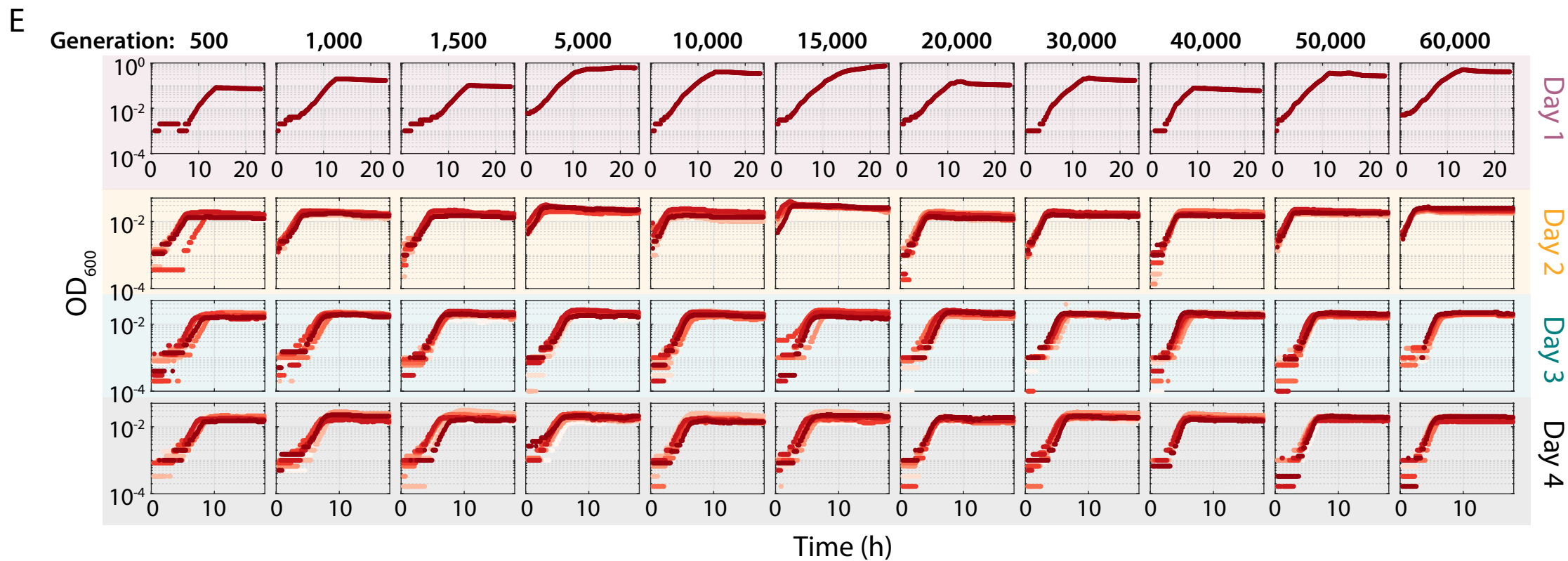

Supplement: FIG S2 [file mBio.01378-20-sf002.pdf]

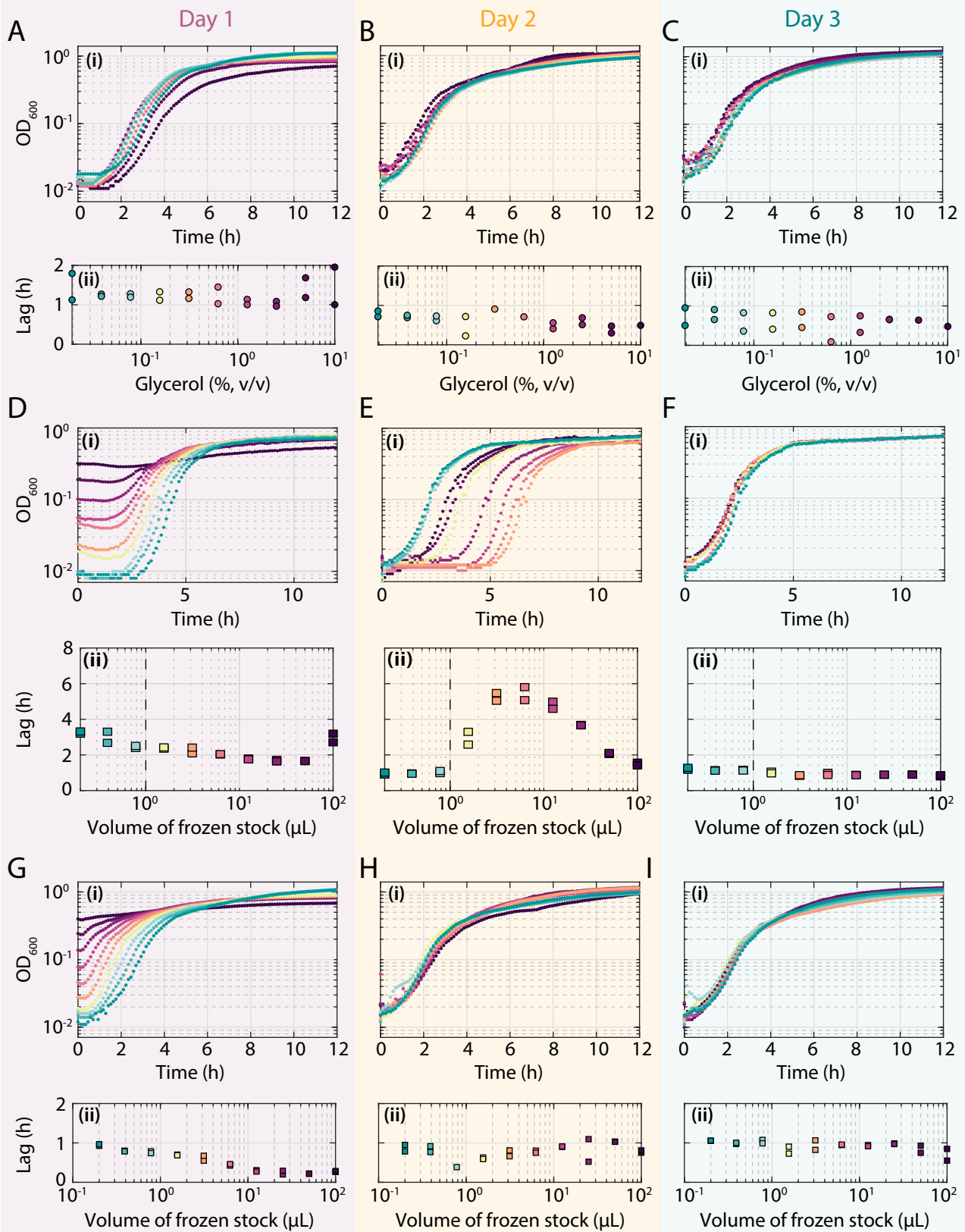

Supplement: FIG S3 [file mBio.01378-20-sf003.pdf]

A

Day 2

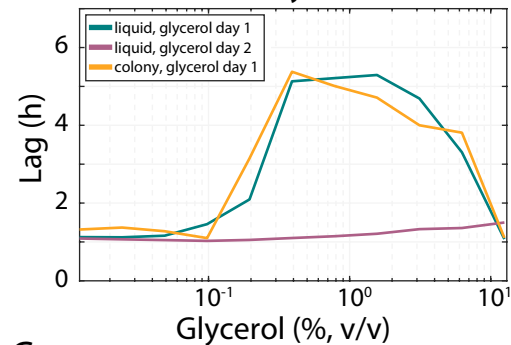

B

Day 3

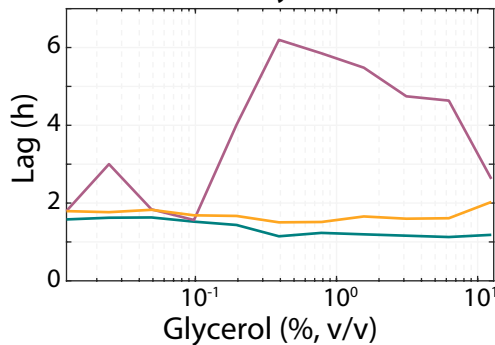

C

Day 2

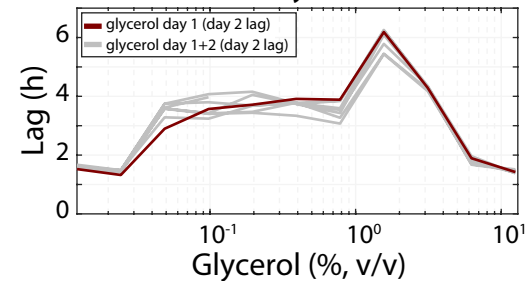

D

LB+glycerol to LB transition

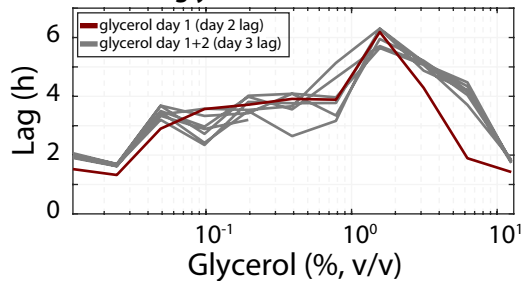

Supplement: FIG S4 [file mBio.01378-20-sf004.pdf]

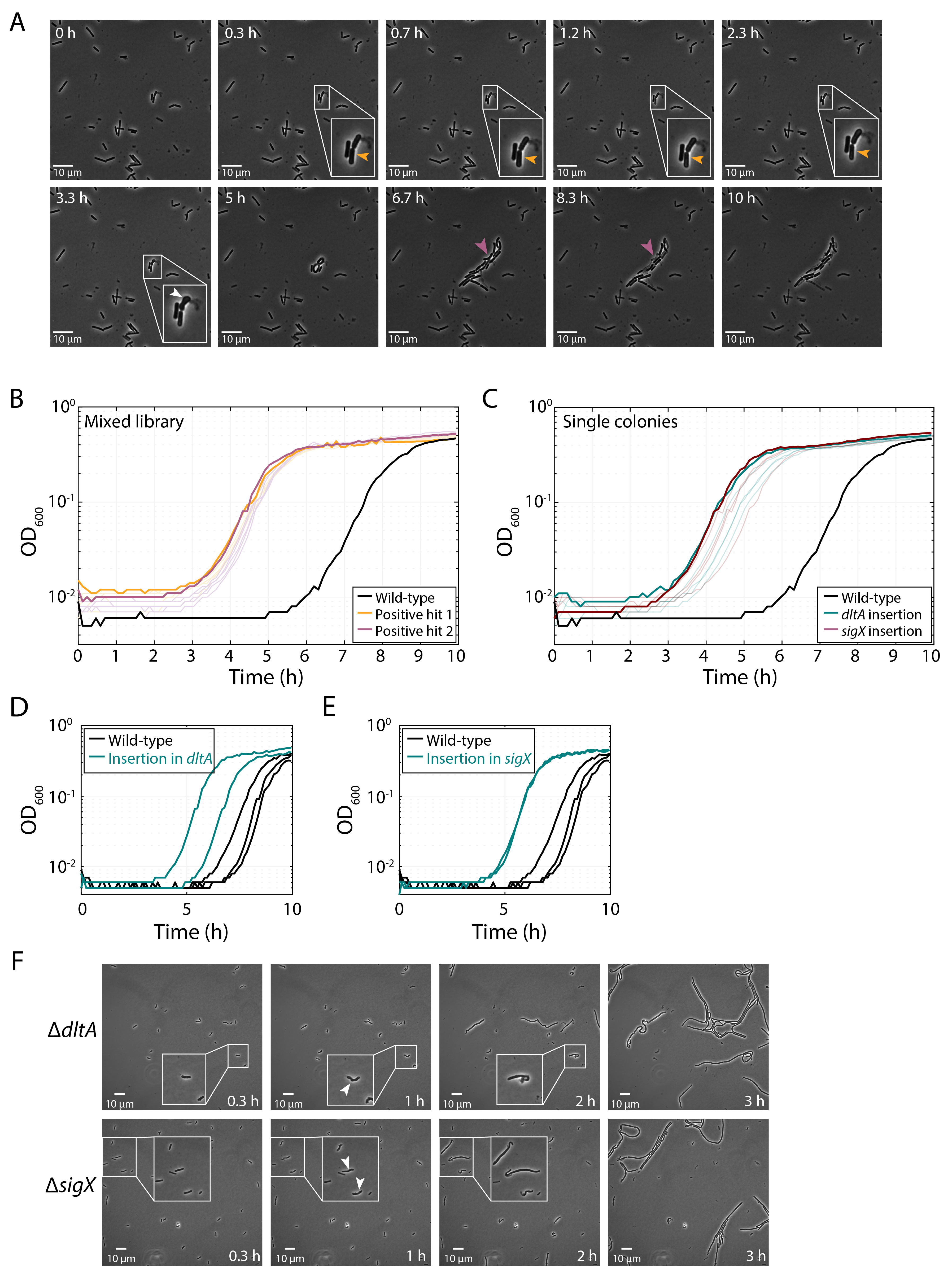

Supplement: FIG S5 [file mBio.01378-20-sf005.jpg]

A

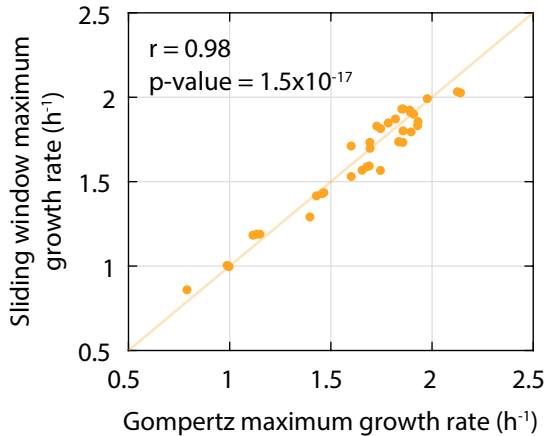

B

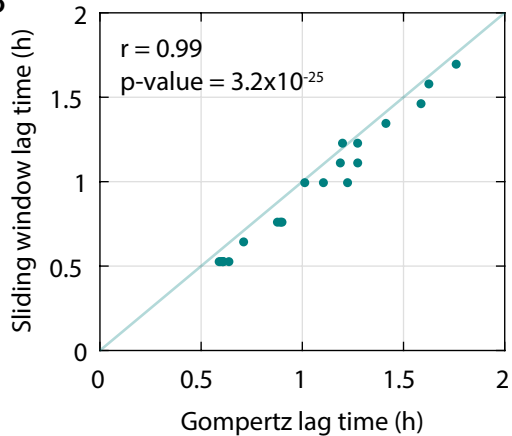

Supplement: FIG S6 [file mBio.01378-20-sf006.pdf]
