# Supplementary material for: Environmental and Physiological Factors Affecting High-Throughput Measurements of Bacterial Growth
Source: mBio. 2020 Oct 20;11(5):e01378-20. doi: 10.1128/mBio.01378-20 (PMC7587430; doi:10.1128/mBio.01378-20)
Supplement: TABLE S1 [file mBio.01378-20-st001.docx]

| **Strain designation** | **Alternative name** | **Genotype or source name** | **Reference, source, or both** |
| --- | --- | --- | --- |
| EA32 | MG1655 | *E. coli* K-12: F^–^ λ^–^ *ilvG*^–^ *rfb-50* *rph-1* | KC Huang’s lab |
| EA33 | REL606 | *E. coli* B: F^–^, *tsx*-467(Am), *araA*^–^, lon^–^, *rpsL*227(*strR*), *hsdR*^–^, [*mal*^+^](*LamS*) | [37], Richard Lenski’s lab |
| EA34 | Ara–1 0.5k | LTEE strain REL762B | [41], Richard Lenski’s lab |
| EA35 | Ara–1 1k | LTEE strain REL964B | [41], Richard Lenski’s lab |
| EA36 | Ara–1 1.5k | LTEE strain REL1068B | [41], Richard Lenski’s lab |
| EA37 | Ara–1 2k | LTEE strain REL1164A | [41], Richard Lenski’s lab |
| EA38 | Ara–1 5k | LTEE strain REL2179A | [41], Richard Lenski’s lab |
| EA39 | Ara–1 10k | LTEE strain REL4536A | [41], Richard Lenski’s lab |
| EA40 | Ara–1 15k | LTEE strain REL7177A | [41], Richard Lenski’s lab |
| EA41 | Ara–1 20k | LTEE strain REL8593A | [41], Richard Lenski’s lab |
| EA42 | Ara–1 30k | LTEE strain REL10391 | [41], Richard Lenski’s lab |
| EA43 | Ara–1 40k | LTEE strain REL10938 | [41], Richard Lenski’s lab |
| EA44 | Ara–1 50k | LTEE strain REL11330 | [41], Richard Lenski’s lab |
| EA45 | Ara–1 60k | LTEE strain REL11691 | Richard Lenski’s lab |
| EA46 | 168 | *B. subtilis*: *trpC2* | Carol Gross’s lab |
| **Back-crossed transposon mutagenesis strains** | | | |
| EA47 | *dltA* | 168, Δ*SPB*, Δ*PBSX*, Δ*pBS32*, *dltA*::*pMarA*-*kan* | This work |
| EA48 | *sigX* | 168, Δ*SPB*, Δ*PBSX*, Δ*pBS32*, sigX::*pMarA*-*kan* | This work |
| **Deletions of transposon mutagenesis strains** | | | |
| BKK38500 | Δ*dltA* | 168, *dltA::kan* | [24] |
| BKK23100 | Δ*sigX* | 168, *sigX::kan* | [24] |
| EA49 | Δ*dltA* | 168, *dltA::kan* | This work |
| EA50 | Δ*sigX* | 168, sigX*::kan* | This work |
